# Practice recommendation for measuring washout rates in ^123^I-BMIPP fatty acid images

**DOI:** 10.1007/s12149-023-01863-8

**Published:** 2023-09-11

**Authors:** Kenichi Nakajima, Hideyuki Miyauchi, Ken-ichi Hirano, Shinichiro Fujimoto, Michitomo Kawahito, Takashi Iimori, Takashi Kudo

**Affiliations:** 1 Department of Functional Imaging and Artificial Intelligence, Kanazawa University, 13-1 Takara-Machi, Kanazawa, 920-8640 Japan; 2 Department of Cardiovascular Medicine, Chiba University Graduate School of Medicine, Chiba, Japan; 3 Department of Triglyceride Science, Graduate School of Medicine, Osaka University, Osaka, Japan; 4 Department of Cardiovascular Biology and Medicine, Juntendo University Graduate School of Medicine, Tokyo, Japan; 5 Department of Cardiology, Shizuoka City Shizuoka Hospital, Shizuoka, Japan; 6 Department of Radiation Technology, Chiba University Hospital, Chiba, Japan; 7 Department of Radioisotope Medicine, Atomic Bomb Disease and Hibakusha Medicine Unit, Atomic Bomb Disease Institute, Nagasaki University, Nagasaki, Japan

**Keywords:** Nuclear medicine imaging, Fatty acid metabolism, Imaging procedure, Single-photon emission computed tomography, Washout rate

## Abstract

The purpose of this practice recommendation is to specifically identify the critical steps involved in performing and interpreting ^123^I-β-methyl-iodophenyl-pentadecanoic acid (BMIPP) single-photon emission computed tomography (SPECT) and measurement of washout rate (WR) from the heart. This document will cover backgrounds, patient preparation, testing procedure, visual image interpretation, quantitation methods using planar and SPECT studies, and reporting of WR. The pitfall and some tips for the calculation of ^123^I-BMIPP WR are also included. The targets of global and regional WR calculation include ischemic heart disease, cardiomyopathy, heart failure, and triglyceride deposit cardiomyovasculopathy, an emerging rare heart disease.

## Background

Scintigraphy using ^123^I- 15-(4-iodophenyl)-3(R,S)-methylpentadecanoic acid (^123^I-BMIPP) is generally regarded as a method of assessing fatty acid images in nuclear medicine. Most basic and research studies of ^123^I-BMIPP have been conducted in Japan. Scintigraphic images are usually acquired at 20 min after ^123^I-BMIPP injection, and their roles in ischemic memory imaging and perfusion-metabolic mismatch are established. Hence, patients with acute and subacute phases of coronary artery disease and vasospastic angina are often assessed by ^123^I-BMIPP imaging [1–4]. A switch from fatty acid to glucose metabolism has been recognized in hypometabolic areas of ^123^I-BMIPP [5]. A ^123^I-BMIPP defect is also useful for assessing patients with ischemic heart disease and those on hemodialysis who have end-stage renal failure [6–8]. The Japan Circulation Society has summarized practice guidelines for ^123^I-BMIPP imaging to determine the diagnosis and prognosis of chronic coronary artery disease (CAD) as it has proven effective and useful [9]. Planar and single-photon emission computed tomography (SPECT) images acquired soon after an intravenous injection of ^123^I-BMIPP is now the most prevalent procedure. Fatty acid metabolism and left ventricular contractility have also been simultaneously evaluated using gated SPECT imaging [10].

Clinical evidence of ^123^I-BMIPP washout rates (WRs) determined from early and late images is limited, but, the fundamental kinetics have been explored since the 1990s. The myocardium uptakes ^123^I-BMIPP dependently on adenosine triphosphate, and subsequent kinetic steps involves alpha and beta oxidation and back diffusion [11–15]. Thereafter, ^123^I-BMIPP is retained mainly in the myocardial triglyceride pool, from which it is slowly cleared. Recent research and clinical studies have investigated WRs using images of patients with ischemic heart diseases and cardiomyopathy. The driving force for this trend is to diagnose triglyceride deposit cardiomyovasculopathy (TGCV) [16–19]. A significantly reduced ^123^I-BMIPP WR is listed as essential in the TGCV diagnostic criteria 2020 of the Research and Development on Intractable Disease by the Japanese Ministry of Labour and Welfare [20], and clinical evidence has accumulated about the value of TGCV images [21–28]. However, data acquisition, analysis, and display methods appropriate for quantifying ^123^I-BMIPP WRs have not been sufficiently investigated [29, 30]. Therefore, this practice recommendation aimed to provide standard procedures for data acquisition and analysis for calculating ^123^I-BMIPP clearance or washout from the heart.

## Radiopharmaceuticals and mechanism of accumulation

The clinical indication for ^123^I-BMIPP scintigraphy is to diagnose cardiac diseases based on fatty acid metabolism. An intravenously injected dose of 74–148 MBq can be adjusted according to age and body weight of patients.

The accumulation of ^123^I-BMIPP in the heart reflects fatty acid metabolism. Cardiomyocytes uptake ^123^I-BMIPP in a concentration gradient, then cluster of differentiation (CD)36 facilitates the transport of long-chain fatty acids, which are moved to the triglyceride pool via BMIPP-CoA. Some ^123^I-BMIPP is transferred to mitochondria, but most of it is retained in the myocardium due to a methyl group at the beta position. This metabolic feature of retention is convenient for SPECT imaging.

## Procedures for ^123^I-BMIPP imaging

### Patient preparation

Patients are required to fast for at least 6 h (6−12 h) before undergoing image acquisition. Water intake is allowed.

### Procedures for imaging

Table 1 shows SPECT or SPECT-CT ^123^I-BMIPP imaging procedures that can be selected according to the nuclear medicine specialty. Patients are intravenously injected with 74–148 MBq ^123^I-BMIPP, then planar and SPECT images are, respectively, acquired at 20 (early) and 180–210 (late) minutes later.

**Table 1:** Methods for ^123^I-BMIPP imaging

Imaging	General procedures
Patient preparation	Fasting at least 6 h (6 − 12 h) except for water intake
Scan	Rest scan
Administered dose	74‒148 MBq; intravenous injection
Time to imaging	Early phase (20 min after injection); SPECT and planar imaging
	Late phase (180‒210 min after injection); SPECT and planar imaging

Imaging	General parameters
Field of view	Cardiac or chest
Image type	Cardiac or chest SPECT and planar
Position	Supine
Energy window	159 keV ± 10%
Matrix	Planar, 256 × 256; SPECT, 128 × 128; at least 64 × 64
Pixel size	4.0‒6.0 mm

Planar imaging	Specific parameters
Views	Anterior
Image duration	300 s
Magnification	× 1

SPECT imaging	Specific parameters
Angular range	Recommended, 180°; optional, 360°
Detector configuration	Recommended, 90°; optional, 180°
ECG gating	Non-gated image for WR calculation; 16 gates (optional 8 gates) for ECG-gated acquisition
View angle	4.0°‒6.0°, step and shoot
Number of views	30‒120 s (adjusted depending on projections)
Time per stop	30‒60 s (adjusted depending on projections)
Magnification	× 1.5 (adjusted for scinticamera field of view and patients’ stature)

Imaging parameters can differ among camera vendors, therefore appropriate adjustments can be made within those recommended above

*ECG* electrocardiography, *WR* washout rate

### Data acquisition

A ^123^I-specific or low-medium energy collimators are needed because ^123^I emits 159 keV gamma rays by electron capture. The energy for data acquisition is centered at 159 keV with a 20% (± 10%) or 15% (± 7.5%) window. Despite low-energy collimators, caution is required regarding increased scatter and septal penetration especially from 529 keV gamma rays.

### Notes for examinations

- Fasting is required before examinations. No food is allowed for at least 6 h (6 − 12 h) before examination except for water intake to avoid influence of foods on myocardial ^123^I-BMIPP uptake and washout rate [31].
- Patient motion artifacts should be avoided.
- Radioisotope leakage during intravenous injection should be avoided.
- Patients must be carefully and precisely positioned for early and late image acquisition.
- Data acquisition protocols for early and late image acquisition should be identical.

### Electrocardiography (ECG)-gated data acquisition

Non-gated images should be used to calculate WRs because ECG-gated images are influenced by rejected arrhythmias.

### SPECT image analysis

Short-, vertical long-, and horizontal long-axis images are generated, and the regional distribution of ^123^I-BMIPP and WRs can be evaluated from early and late polar maps.

### Notes for imaging

- Single-, rather than dual-radionuclide imaging is recommended for ^123^I-BMIPP WR calculations.
- Dual-radionuclide assessment with ^123^I-BMIPP and ^201^Tl might affect the accuracy of WR calculations. Crosstalk between energy peaks of ^123^I (159 keV) and ^201^Tl (Hg-X 71–80 keV, 167 keV [10%]) can be corrected using various methods recommended by individual camera suppliers. However, when dual-nuclide acquisition is required, the effects of crosstalk should be assessed in advance.
- As attenuation and scatter correction might affect the accuracy of WR calculation, these should not be corrected at present because methods vary among equipment vendors. Thus, the accuracy of individual methods requires determination.
- Washout rates can be calculated using images with a cardio-centric configuration acquired by cameras with cadmium-zinc-chloride (CZT) detectors with high resolution and high sensitivity. However, the reliability of WR calculations requires further investigation due to limited experience.

## Visual interpretation

**Fig. 1:**
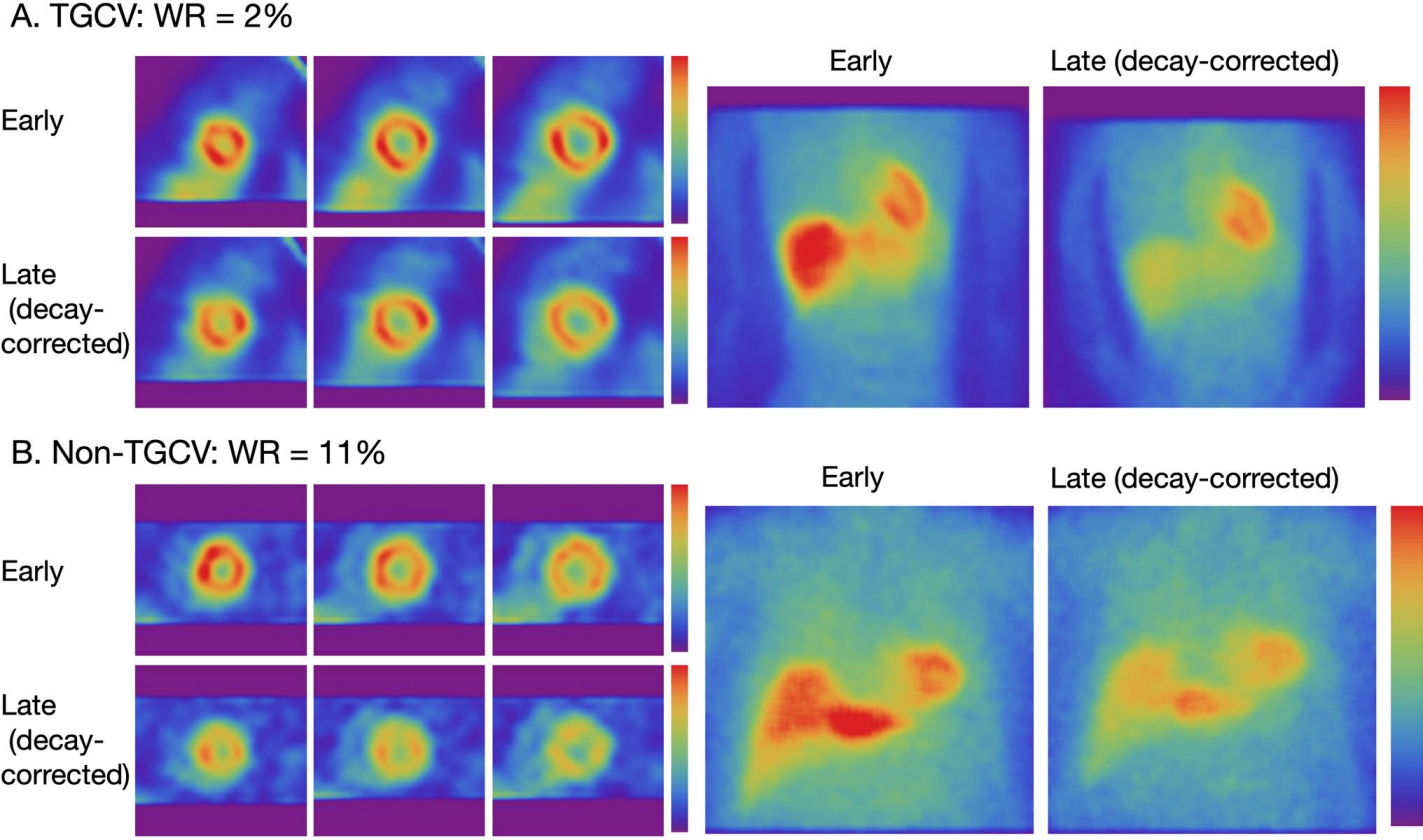
Three short-axis SPECT slices and anterior planar images of patients. **A** Patient with WR 2% and clinical diagnosis of TGCV. **B** Patient with WR 11%. Color scale of late images was corrected for time decay between early and late imaging. This can be achieved either by multiplying counts derived from late images by decay correction factor to the late image or modifying the maximum count in the late image adjusted for time decay. Correction for time decay led to similar heart counts between early and late images in patient A, whereas heart count was decreased in late image in patient B. *TGCV* triglyceride deposit cardiomyovasculopathy, *WR* washout rate

**Fig. 2:**
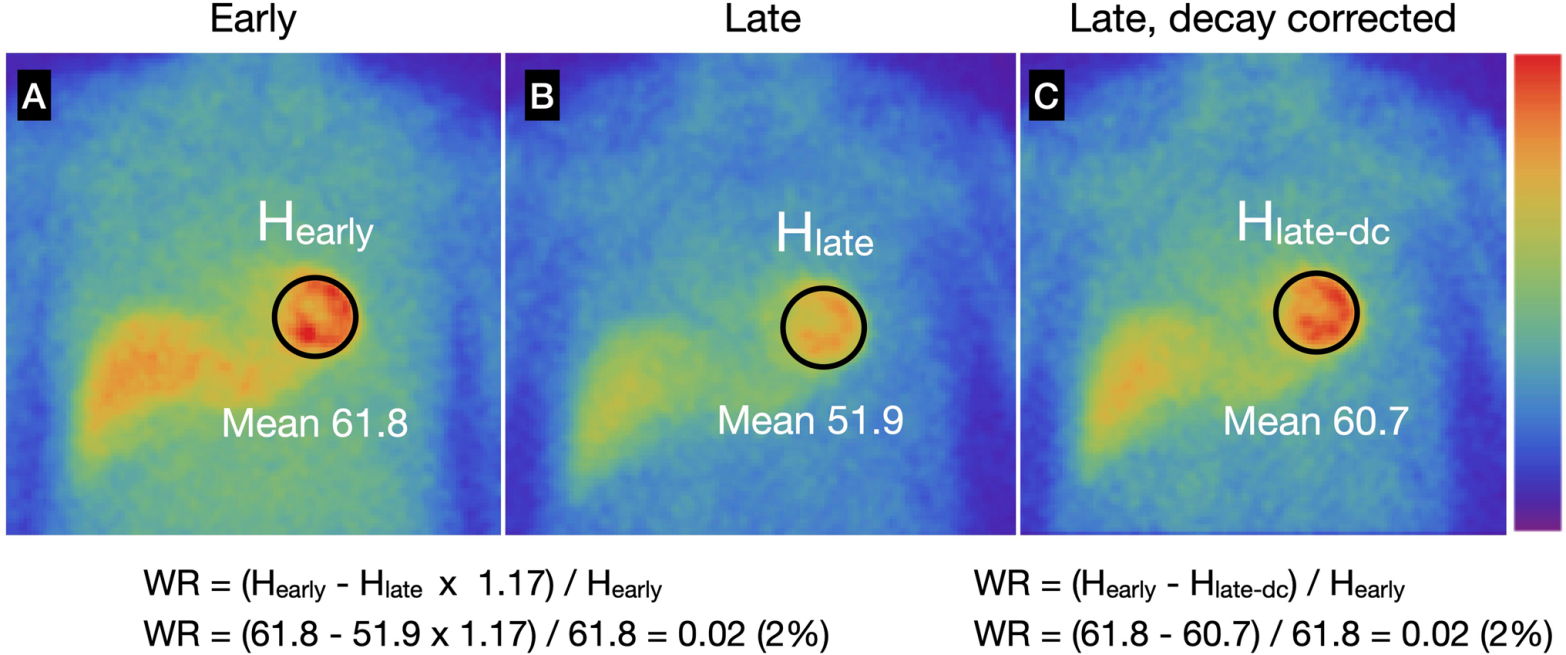
Examples of washout rates calculated using early (**A**), late (**B**) and decay-corrected late (**C**) planar images. The WR can be calculated from the average heart counts. Background correction is not applied. H_early_, early heart count; H_late_, late heart count; H_late-dc_, late heart count after decay correction. When interval between early and late images is 3 h (physical half-life of ^123^*I* = 13.2 h), calculated decay correction factor is 1.17 (Table 2). *WR*, washout rate

Cardiac accumulation of ^123^I-BMIPP is confirmed using anterior planar images. Time-dependent count decay should be corrected because ^123^I-BMIPP is usually washed out from the heart within 4 h, which is within the timeframe when early and late images are acquired. Myocardial washout can then be interpreted visually using the same scale that was displayed after decay correction (Figs. 1 and 2).

Metabolic defects and the homogeneity of ^123^I-BMIPP distribution can then be evaluated using standard short-, vertical long-, and horizontal long-axis images after standard reconstruction.

### Notes for interpretation

**Fig. 3:**
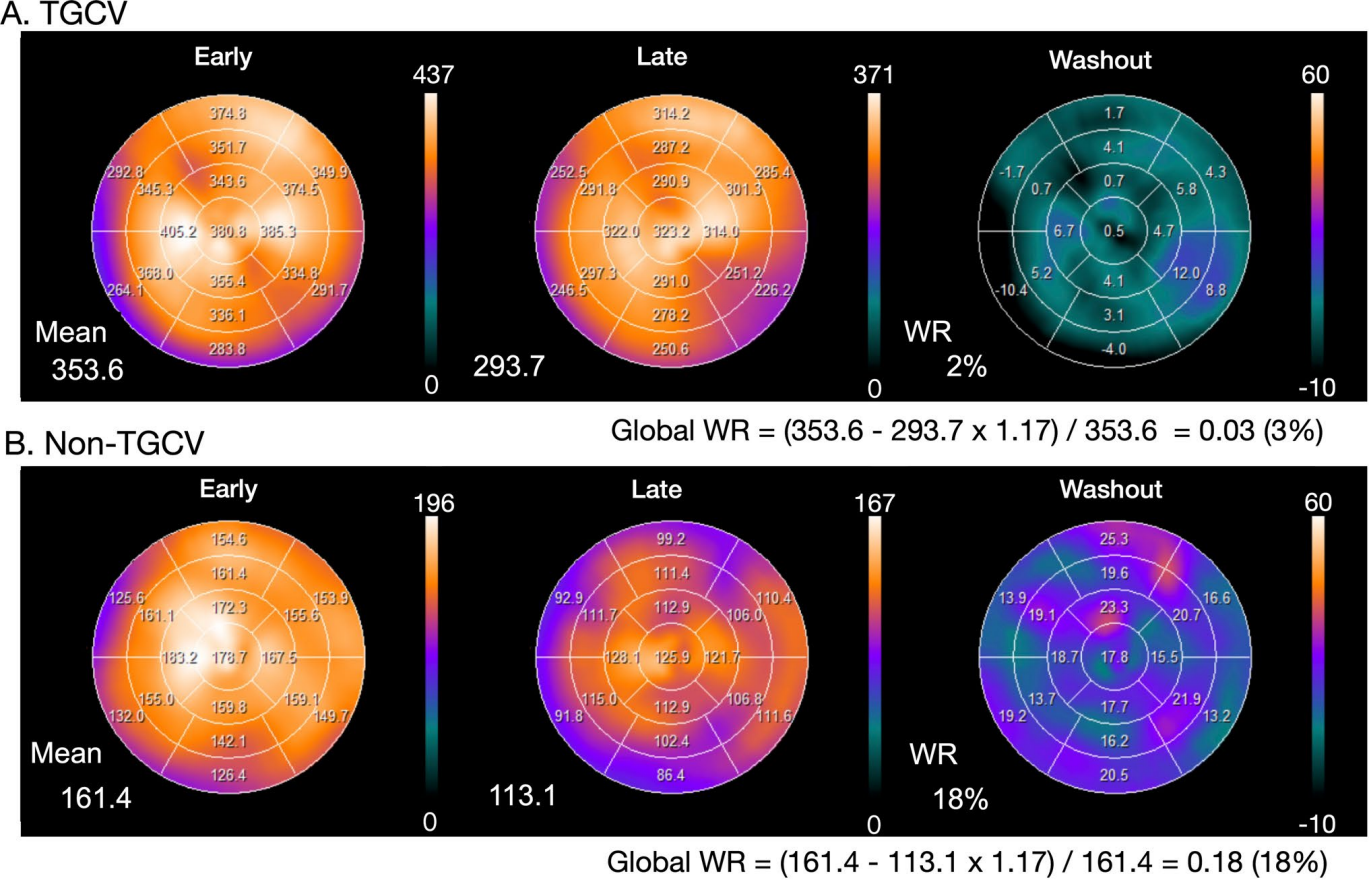
Examples of WRs in SPECT polar maps. **A** Clinical diagnosis of TGCV with WR 3%. **B** Coronary artery disease with WR 18%. Late image is shown with color scale in which maximum count is multiplied by a time-decay correction factor of 3 h (1.17). Global WR can be calculated by defining WR (Eq. 1) after decay correction. Compared with average of pixel-based WR (left lower corner of WR polar map, 2%), global WR (3%) was in agreement when regional WR values were homogeneous. However, averaged pixel-based WR might be influenced by misaligned early and late slices or defective regions. *WR* washout rate

- Time-dependent decay can be calculated as 0.5^ (duration between early and late images/13.2 [h]). For example, 3-h decay can be corrected by multiplying counts derived from late images by 1.17 (Table 2).
- Washout is invisible when early and late SPECT images are each displayed separately on a scale of 0‒100%. However, myocardial washout can be easily interpreted visually when the display range is adjusted for decay correction in the late image. Figures 2 and 3 show late images for planar and SPECT studies, respectively.

**Table 2:** Physical decay of ^123^I and decay correction factors

Time (h)	Decay	Decay correction factor
2.5	0.88	1.14
2.75	0.87	1.16
3.0	0.85	1.17
3.25	0.84	1.19
3.5	0.83	1.20
3.75	0.82	1.22
4.0	0.81	1.23

The half-life of ^123^I is 13.2 h

## Quantitation of washout rates

Washout rates (%) can be calculated for planar and SPECT images using polar maps as:

1 $$\frac{{{\text{Early heart count}} - {\text{Late heart count}} }}{{\text{Early heart count}}} \times 100,$$

where late heart counts are corrected for ^123^I decay with a half-life of 13.2 h.

**Fig. 4:**
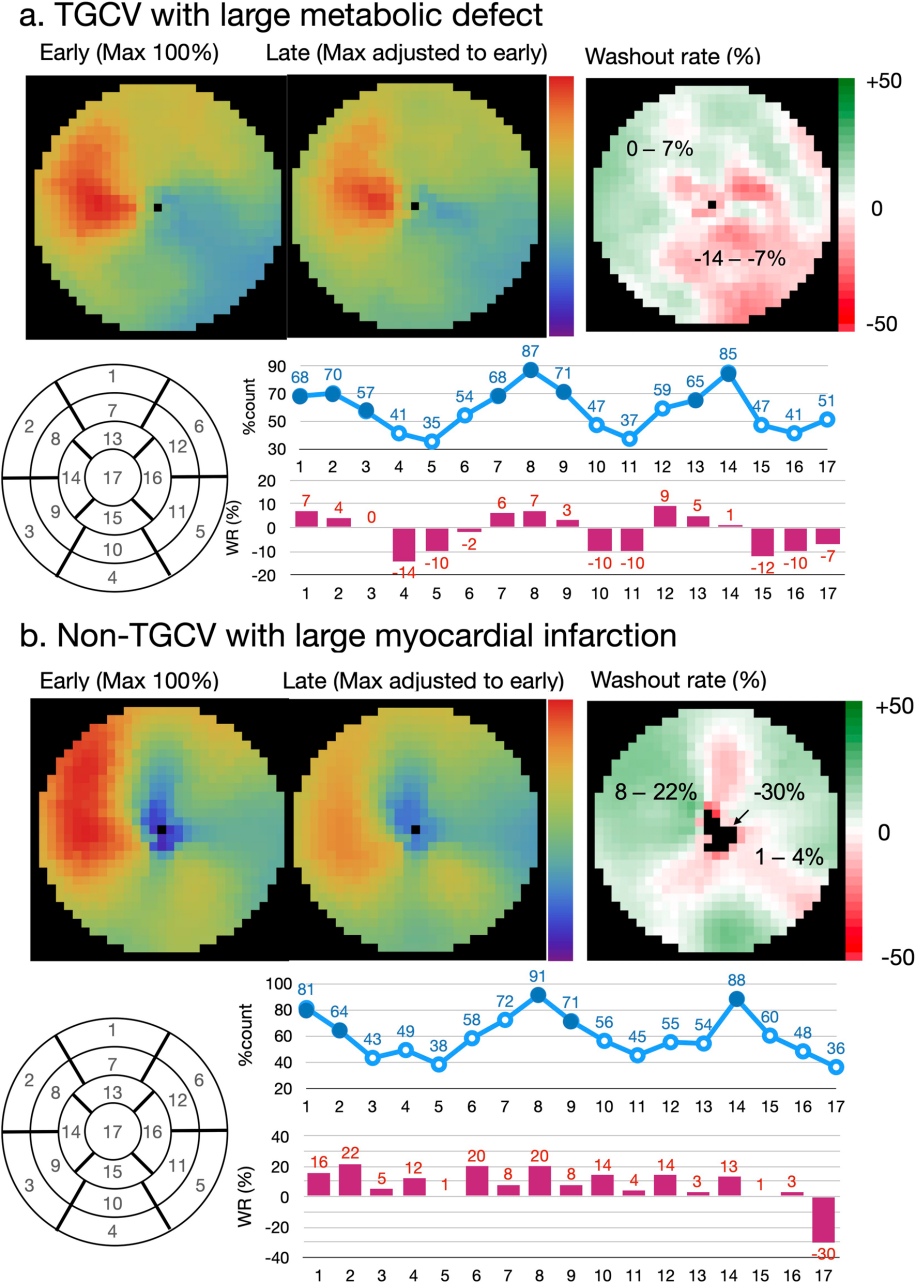
Examples of washout rates calculated from images of patients with metabolic defects. Line graph (blue), percent-counts (%) in early myocardial count using a 17-segment model. Solid circles indicate segments with preserved accumulation. Bar graph (red) shows regional WR per segment. Patient A **a** was clinically diagnosed with TGCV. Average anteroseptal WR in metabolically preserved segments was 4.1%. WR: (mean early count–mean late count)/mean early count was 1%; averaged pixel based,  − 3%; defective region (inferolateral–apex)  − 14 to  − 7%; anteroseptal (blue circle), 0–7%. Patient B **b** had old myocardial infarction and three-vessel disease. WR: (mean early count–mean late count)/mean early count was 9%; averaged pixel based, 0%; infarction (inferolateral), 1–4% and apex, − 30%; anteroseptal (blue circle), 8–22%. Average anteroseptal WR in metabolically preserved segments was 15.8% (13–22%) Adapted from reference [29]. *TGCV* triglyceride deposit cardiomyovasculopathy, *WR* washout rate

1. Calculating WRs using a planar image: A circular (elliptical or heart-shaped) region of interest (ROI) is set on the heart, and the WR is calculated as average heart counts on early and late images (Fig. 2). The cardiac ROI should be placed on the heart and should not extend outwards. A background ROI is not required.
2. Calculating WRs using SPECT polar maps: Washout rates are calculated as average counts according to Eq. 1. after early and late counts are averaged (Fig. 3). Appropriate selections of slice ranges at the base and apex are important to create a polar map. Early and late images that can be misaligned when basal slices near the valve plane are selected could result in inaccurate WR results. This method can be used for TGCV, ischemic heart diseases, cardiomyopathy, and heart failure.
3. Regional and segmental WR calculation using SPECT polar maps: Commercial software can calculate WRs using three, five, or 17 segments, or three regions. These algorithms can be applied for example, to compare WRs in normal regions with those that have reduced metabolic activity between normal and ischemic myocardia, and among three coronary artery territories (Fig. 4).

### Notes for WR calculation

- The processing range of basal and apical slices must be carefully determined when the WR is calculated using ^123^I-BMIPP SPECT. A long-axis image can be used as a reference for the selection of slice ranges, and a polar map display of WR is convenient for checking outlier WR values. One algorithm calculates WRs using average counts in early and late images, and another averages pixel-based WRs on polar maps [29, 30]. Although the results of the two methods generally agree when patients are defect-free, large metabolic defects and misaligned settings of the slice range could affect pixel-based average WRs.
- Background subtraction using a mediastinal ROI could cause fluctuations in WR and is not recommended for calculating WRs from planar images [29].
- The scale of the polar map can be count-based after decay is corrected on late polar maps. This is preferable to using a percentage scale (0‒100%) to confirm differences in counts between early and late images. Washout rates can be calculated using counts averaged from early and late polar maps and Eq. 1 (Fig. 3).
- When patients have large metabolic defects due to previous myocardial infarction or severe fibrosis, defective segments can decrease regional WRs, and misaligned defect segments could cause fluctuations in regional WRs. Outlier WRs that are calculated regionally from defect segments should be excluded (Fig. 4) [29]. Global WRs should not be misinterpreted by regionally deranged metabolic activity since a decreased WR is critical for a diagnosis of TGCV. In addition to global WRs, regional distribution should be carefully interpreted when calculating WRs.
- Washout rates can be calculated from averaged early and late counts derived from summed short-axis images of base to apical slices [29].
- Time decay correction factors can be calculated as (Table 2):
  $$1/0.5^{ \wedge } ({\text{elapsed}}\;{\text{time}}\;{\text{between}}\;{\text{early}}\;{\text{and}}\;{\text{late}}\;{\text{images}}/13.2[{\text{h}}]).$$

## Radiation exposure

The respective absorbed radiation doses (mGy/MBq) according to the Committee on Medical Internal Radiation Dose (MIRD) for the heart, ovaries, testes, and whole body are 0.057, 0.011, 0.0076, and 0.010. The effective dose can be calculated using 0.016 mSv/MBq for adult (International Commission on Radiological Protection [ICRP] Publication 128, Annals of the ICRP 2015; 44, No. 2S).

## Data Availability

Available at https://www.icrp.org/publication.asp?id=ICRP%20Publication%20128.
