# Correction: Practice recommendation for measuring washout rates in ^123^I-BMIPP fatty acid images

**DOI:** 10.1007/s12149-023-01880-7

**Published:** 2023-10-14

**Authors:** Kenichi Nakajima, Hideyuki Miyauchi, Ken-ichi Hirano, Shinichiro Fujimoto, Michitomo Kawahito, Takashi Iimori, Takashi Kudo

**Affiliations:** 1 Department of Functional Imaging and Artificial Intelligence, Kanazawa University, 13-1 Takara-Machi, Kanazawa, 920-8640 Japan; 2 Department of Cardiovascular Medicine, Chiba University Graduate School of Medicine, Chiba, Japan; 3 Department of Triglyceride Science, Graduate School of Medicine, Osaka University, Osaka, Japan; 4 Department of Cardiovascular Biology and Medicine, Juntendo University Graduate School of Medicine, Tokyo, Japan; 5 Department of Cardiology, Shizuoka City Shizuoka Hospital, Shizuoka, Japan; 6 Department of Radiation Technology, Chiba University Hospital, Chiba, Japan; 7 Department of Radioisotope Medicine, Atomic Bomb Disease and Hibakusha Medicine Unit, Atomic Bomb Disease Institute, Nagasaki University, Nagasaki, Japan

**Correction: Annals of Nuclear Medicine** 10.1007/s12149-023-01863-8

The article “Practice recommendation for measuring washout rates in ^123^I-BMIPP fatty acid images”, written by Kenichi Nakajima, Hideyuki Miyauchi, Ken-ichi Hirano, Shinichiro Fujimoto, Michitomo Kawahito, Takashi Iimori, Takashi Kudo, was originally published electronically on the publisher’s internet portal on 11 September 2023 without open access. With the author(s)’ decision to opt for Open Choice the copyright of the article changed on 2 October 2023 to © The Authors 2023 and the article is forthwith distributed under a Creative Commons 4.0 International License, which permits use, sharing, adaptation, distribution and reproduction in any medium or format, as long as you give appropriate credit to the original author(s) and the source, provide a link to the Creative Commons licence, and indicate if changes were made. The images or other third party material in this article are included in the article’s Creative Commons licence, unless indicated otherwise in a credit line to the material. If material is not included in the article’s Creative Commons licence and your intended use is not permitted by statutory regulation or exceeds the permitted use, you will need to obtain permission directly from the copyright holder. To view a copy of this licence, visit http://creativecommons.org/licenses/by/4.0/.

The original article has been updated.

